# The Proportion of Student Tuberculosis Cases and Treatment Outcome at Jimma University Medical Center: 5-Year Retrospective Study (11 Sep. 2010–10 Sep. 2015)

**DOI:** 10.1155/2019/4597154

**Published:** 2019-03-03

**Authors:** Hiwot Tibebu, Habtemu J. Hebo

**Affiliations:** ^1^Medicine and Health Officer Coordinating Office, Medical Science Faculty, Institute of Health, Jimma University, P.O. Box 378, Jimma, Ethiopia; ^2^Department of Epidemiology, Public Health Faculty, Institute of Health, Jimma University, P.O. Box 378, Jimma, Ethiopia

## Abstract

**Background:**

University students are highly congregated at classroom and residence (dormitory) which offer a special risk to exposure and transmission of tuberculosis. In Ethiopia, the number of students joining universities is increasing from time to time though infrastructure of the universities has not kept pace with this increment. However, compiled reports on the magnitude and trend of tuberculosis in the higher education institutions of Ethiopia are limited.

**Objective:**

This study was designed to determine the five years (Sep. 2010 to Sep. 2015) trend of the proportion of student tuberculosis cases and treatment outcome at Jimma University Medical Center.

**Methods:**

A cross-sectional study was conducted at Jimma University Medical Center. A total of 347 students from Jimma University who were registered and treated at Tuberculosis Clinic of Jimma University Medical Center were included in this study. Data were collected by record review using checklist prepared in English. Data were entered into EpiData and cleaned and analyzed by SPSS 20.

**Results:**

The mean proportion of student tuberculosis cases among total adult tuberculosis cases was 29.71% (347/1168). The mean proportion of student tuberculosis cases among the total students enrolled was nearly 0.38% (347/92,004). More than three-fourths (76.37% (265/347)) were male. Pulmonary tuberculosis accounted for 72.62% (252/347) and 54.40% (137/252) of these were smear-positive. Eight cases were positive for Human Immunodeficiency Virus. More than four-fifths (281/347) were new cases. The highest proportion (37.62%) was observed in 2010/11 while the lowest (12.03%) was observed in 2012/13. The proportion of tuberculosis dramatically decreased in the third year and significantly increased again in the last two years. Regarding treatment outcome, 98.75% (316/347) had successful treatment outcome (61.71% treatment completed; 38.29% cured). Four cases were defaults and there was not any death.

**Conclusion:**

The five years' mean proportion of student tuberculosis cases among the total students enrolled was high in this study. However, the treatment success rate was better than the report of previous studies. Therefore, governmental and nongovernmental organizations concerned with tuberculosis must consider universities as focal points for the prevention and control of tuberculosis in Ethiopia.

## 1. Background

Tuberculosis (TB), caused by the bacillus* Mycobacterium tuberculosis complex*, is one of the leading infectious diseases and health burden in the world [1]. Despite the availability of highly efficacious treatment for decades, tuberculosis remains a major global public health problem infecting one-third of the world population and putting them at risk of developing active disease during their lifetime [2–4]. Today, tuberculosis incidence is 100 times higher than the elimination target for 2050 which is an incidence of less than one case per one million population [4]. The highest incidence rates are in Africa where high rates of Human Immunodeficiency Virus (HIV) and malnutrition weaken immune systems and fuel the spread of the disease. Tuberculosis is also the second leading cause of death from infectious diseases worldwide, after HIV [5].

Ethiopia ranks third in Africa and eighth among the 22 highest tuberculosis burdened countries in the world that collectively account for 80% of tuberculosis cases [3, 6]. The prevalence of all forms of tuberculosis is estimated at 261 per 100,000 people, leading to an annual mortality rate of 64 per 100,000 people. The incidence rate of all forms of tuberculosis is estimated at 359 per 100,000 people, while the incidence rate of smear-positive tuberculosis is 108 per 100,000 people. The tuberculosis case detection rate, treatment success rate, and cure rate are 74%, 82.5%, and 67%, respectively [6].

Tuberculosis has the potential to affect all people regardless of sex and age; different groups of people have a variable chance to contract the infection and to develop the disease. It predominantly occurs among young adults (between the ages of 15 and 54) where approximately 75% of all tuberculosis cases arise coinciding with people's most productive years [4, 7]. In places where contact with infectious individuals occurs, risk for acquiring tuberculosis infection is increased [8]. Crowding and poor ventilation can increase the risk of transmission in such settings. Those at risk for increased exposure include residents and employees of congregated settings.

University students are highly congregated at classroom and residence (dormitory) which offer a special risk to exposure and transmission of tuberculosis [9]. University students also are usually 18-23 years old, characterized by rapid physical development and endocrine instability, and reports have revealed that the incidence of pulmonary tuberculosis (PTB) starts to rise greatly during this period [4, 10].

In Ethiopia, the number of students joining universities/colleges is increasing from time to time though infrastructure of the universities/colleges has not kept pace with this increment. This forces students to spend 3-6 years in a crowded living environment where there are more than 20 students per dormitory, 100-200 students per classroom, and 2000-5000 students in a single dining hall who congregate for meals three times a day [4, 11].

Thus, the living conditions of Ethiopian universities could be favorable for tuberculosis transmission. However, compiled reports on the magnitude and trend of tuberculosis in the higher education institutions of Ethiopia are limited. Therefore, this study was designed to determine the five years (Sep. 2010 to Sep. 2015) trend of the proportion of student tuberculosis cases and treatment outcome at Jimma University Medical Center. Thus, the findings of this study could be indications to attract attention toward students at universities for achieving the new post-2015 Global TB Strategy called “END TB strategy.”

## 2. Methods and Materials

### 2.1. Study Setting and Period

This study was conducted at Jimma University Medical Center (JUMC). It is located in Jimma town at around 352 km Southwest of Addis Ababa. As being one of the country's higher referral hospitals, it provides a lot of services to patients per year especially for those living in the Southwestern region of the country. Currently, it is the only teaching and referral hospital in the Southwestern part of the country, providing services for approximately 15,000 inpatient, 160,000 outpatient attendants, 11,000 emergency cases, and 4500 deliveries in a year coming to the hospital from the catchment population of about 15 million people.

JUSH has a total of 861 technical and 587 supportive staffs. Since it is a teaching hospital, it has about 145 residents, 250 medical interns, and 120 health officer interns taking either long term or short term practical attachments. The hospital also has a separate student clinic which offers many services to Jimma University students including health promotion and disease prevention, adolescent and youth reproductive health services, and routine health provision. According to the information obtained from Jimma University's Registrar Office, the estimated total number of undergraduate regular students during the study period was 92,004, of which 26,491 were females. The BCG status of the students was not known. Mantoux or tuberculin skin test (TST) was also not done when the students were entering the university.

### 2.2. Study Design

A cross-sectional study design was used.

### 2.3. Study Population

The study population was all student tuberculosis cases registered and treated at Jimma University Medical Center (JUMC) during the study period (Sep. 11, 2010-Sep. 10, 2015). The inclusion criterion was an undergraduate regular university student with tuberculosis registered and treated at Jimma University Medical Center's Tuberculosis Clinic. However, transfer-out cases were excluded from the denominator for calculating treatment success and default rates.

### 2.4. Sample Size and Sampling Technique

All university student tuberculosis cases registered and treated at JUMC during the study period and that fulfill inclusion criteria were included.

### 2.5. Variables

#### 2.5.1. Dependent Variables

- The proportion of student tuberculosis cases among the total adult tuberculosis cases
- The proportion of student tuberculosis cases among the total students
- Treatment outcome of student tuberculosis cases

#### 2.5.2. Independent Variables

- Age, sex, HIV serostatus, type of TB, smear status, year registered and treated, and treatment category

### 2.6. Data Collection Process

Data were collected by record review using checklist prepared in English language. Two data collectors were assigned to collect data. Before data collection, training was given for data collectors regarding data collection procedure and data quality insurance. A principal investigator supervised the completeness and consistency of filled formats.

### 2.7. Data Processing and Analysis

Data were entered into EpiData and cleaned and analyzed by SPSS 20. Descriptive statistics such as proportion, mean, and standard deviation were calculated. Results were presented in text, tables, and figures.

### 2.8. Ethical Consideration

Ethical clearance was obtained from Ethical Review Board of the Institute of Health, Jimma University. Support letter from Jimma University Student Research Program was submitted to JUMC to obtain permission to use the data for the purpose of this study. All information obtained from the individual record was kept confidential by excluding personal identifiers. Information obtained was also used only for the purpose of this study.

### 2.9. Operational Definition

- *Successful treatment*: the sum of cured and treatment completed cases.

## 3. Results

### 3.1. Sociodemographic and Clinical Characteristics

A total of 347 students from Jimma University who were registered and treated at JUMC's TB Clinic were included in this study. More than three-fourths (265/347) were male. The age of the participants ranged from 18 to 33 years and the majority (202/347) were in the age group 21-26 years. The median age of participants was 21 (IQR = 20-22) years. Pulmonary tuberculosis accounted for 72.62% (252/347) and 54.37% (137/252) of these were smear-positive. There was not any disseminated tuberculosis recorded. Eight cases were positive for HIV. More than four-fifths (281/347) were new cases, 18.73% were transfer-in (TI), and 1 was relapse (Table 1).

### 3.2. Proportion, Trend, and Treatment Outcome of Student TB Cases

The mean proportion of student TB cases among total adult TB cases on follow-up over five years study period was 29.71% (347/1168). The mean proportion of student TB cases among total students enrolled was nearly 0.38% (347/92,004). The mean proportion of male student TB cases among total male students enrolled was nearly 0.40% (265/65,513), whereas the mean proportion of female student TB cases among total female students enrolled was nearly 0.31% (82/26,491) (Table 2).

**Figure 1:**
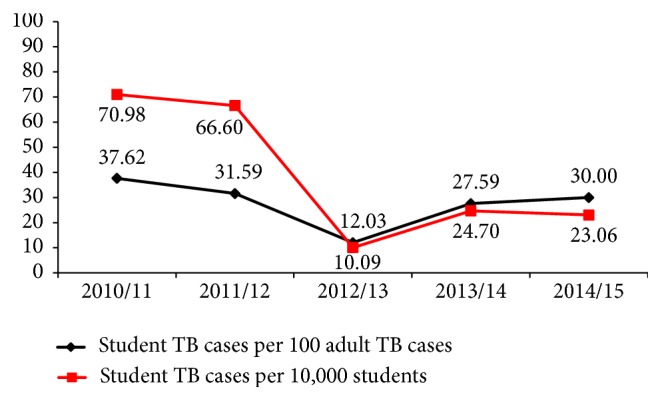
The trend of proportions of student TB cases among total adult TB cases and among total students, JUMC, 11 Sep. 2010-10 Sep. 2015.

The highest student TB proportion (37.62%) was seen in the period 11 Sep. 2010-11 Sep. 2011 while the lowest (12.03%) was observed in the period 11 Sep. 2012-10 Sep. 2013. The proportion of student TB cases among the total adult TB cases dramatically decreased in the third year and significantly increased again in the last two years though it was not as worrying as in the first and second years. The proportion of student TB cases among total students enrolled had a similar pattern to the proportion of student TB cases among total adult TB cases, except for the last year when it was observed to be decreased (Figure 1).

Regarding treatment outcome, 316 (98.75%) had successful treatment outcome (61.71% treatment completed and 38.29% cured) while 4 were defaults and there was not any death.

## 4. Discussion

This analysis had determined the proportion of student TB cases and assessed treatment outcomes. Universities tend to be highly congregated settings in classrooms, dormitory, and cafeteria and, thus, provide special opportunities for a lot of people to be exposed to a person with TB. In our analysis, nearly one-third of total adult TB cases on follow-up over five-year study period were student TB cases. The five years' mean proportion of student TB cases among the total students enrolled was nearly 37.72 per 10,000 students. A similar five years' mean proportion of 36.07 per 10,000 students enrolled was reported by a study conducted in Gondar University, northwest Ethiopia [11]. However, it was lower than the five years' mean proportion of 53.50 per 10,000 students enrolled at Sidist Kilo campus of Addis Ababa University (AAU), central Ethiopia [4]. It was also too much lower than the five years' mean proportion of 107.20 per 10,000 students enrolled at Adama Science and Technology University (ASTU), central Ethiopia [4]. The proportion of student TB cases among the total students enrolled in each year was higher than the national prevalence during the study period, except for the year 2012/13 which was lower [2, 12–16]. Although the five years' mean proportion was similar to or lower than the report of earlier studies, the inflated proportion in the first two years shows that TB outbreak existed in the university during that period. A health professional working in the TB Clinic for a long time confirmed that there was a TB outbreak among Jimma University students during the indicated period. The national TB prevalence was also the highest (39.78 per 10,000) in 2010 during the study period [12]. However, we could not get any recorded evidence regarding the existence and possible cause of the outbreak. Studies have indicated that students at higher education institutions tend to be exposed to TB due to congested settings in both dormitories and classrooms. It is also possible that TB acquired outside the university campus may get reactivated in students under stress in universities [4]. The outbreak encompassing a total of 24 TB cases was reported from 2009 to 2014 at an educational institution in Oslo, Norway [17]. In China, twenty-one cases of clustered TB in schools had been reported nationwide from January 2009 to June 2013. The average number of patients reported per case was twenty-five, of which fourteen were high school patients, accounting for 66.7% [18].

In the current study, most cases (72.62%) were pulmonary TB (PTB) and more than half of these (54.37%) were smear-positive. The proportion of PTB cases was similar to the report of a study conducted at Gondar University, but the proportion of smear-positive cases was higher in our analysis [11]. The proportion of PTB cases was also slightly higher and significantly higher than the reports from AAU and ASTU, respectively. The proportion of smear-positive cases was also higher than the report from ASTU though it was lower than the report from AAU [4]. This indicates that the risk of transmission was higher in our setup which deserves strong attention to control the cycle of transmission. The higher proportion of smear-positive TB cases clearly showed that a considerable amount of TB was circulating among students implying that students could be the foci of infection not only to their peers but also to the community at large. It should also be noted that occurrence of even a single case of contagious tuberculosis in university/college campus could be the reason for anxiety and concern for students.

Male students constitute a significantly higher proportion of tuberculosis cases in this study. This is in agreement with the report of other studies from Ethiopia [4, 11]. The mean proportion of male student TB cases among total male students enrolled was also higher than the mean proportion of female student TB cases among total female students enrolled. This could be explained by behavioral and immunological factors that contribute to an increased risk of TB acquisition in men [19]. Eight cases were seropositive for HIV indicating the existence of epidemics of HIV in the university.

The proportion of cured cases was almost twice higher than the report of a study conducted in northwest Ethiopia. Treatment completed was also higher in our analysis than the report of a study conducted in northwest Ethiopia. However, the default was lower in our analysis than the report of a study conducted in northwest Ethiopia. In general, the treatment success rate was better in our analysis compared to the report of a study conducted in northwest Ethiopia [11].

The trend of tuberculosis proportion showed a steady decline from the first to the second year and sharply declined and attained the lowest value in the third year of the study period. However, the proportion again sharply rose up in the fourth and fifth years of study period. This differed from the report of a study conducted in central Ethiopia where the trend showed a steady decline from the first to last year of the study period [4].

## 5. Limitations

It should be understood that the proportion observed in this study might actually underestimate the true burden of the disease in university as some cases might have been diagnosed and obtained the directly observed treatment short course (DOTS) service in private health facilities of preference. Another limitation that might have affected the proportion of TB among students is that the total size of students during the study period might not be the real figure. Some students might have left the university through withdrawal, dismissal, transfer-out, or another reason. Others might have joined the university through readmission, transfer-in, or another reason.

## 6. Conclusion

The five years' mean proportion of student TB cases among the total students enrolled was high in this study. Most cases were PTB and the majority was smear-positive PTB cases which might exacerbate the cycle of transmission as observed in disease trend which declined in the first three years and again rose up in the next two years. However, the treatment success rate was better than the report of previous studies. Therefore, governmental and nongovernmental organizations involved in tuberculosis control must consider higher education institutions as focal points for prevention and elimination of tuberculosis in Ethiopia.

Availing regular TB screening program (surveillance) and provision of TB awareness information for all students at entry may contribute to the control and prevention of the problem. Screening and targeted testing for tuberculosis (TB) is a key strategy for controlling and preventing infection in university. Early detection provides an opportunity to promote the health of affected individuals through prompt diagnosis and treatment while preventing potential spread to others. Medicine and Health Science Students in clinical practice should be given priority as they have frequent contacts with patients in the hospital and can bring the problem to other students.

## Figures and Tables

**Table 1:** Characteristics of study participants, JUMC, 11 Sep. 2010-10 Sep. 2015.

Participant's characteristics		Number	Percent
Sex	Female	82	23.63
	Male	265	76.37

Age	≤ 20	126	36.31
	21-25	202	58.21
	> 25	19	5.48

Type of TB	Pulmonary Tuberculosis (PTB)	252	72.62
	Extra Pulmonary Tuberculosis (EPTB)	95	27.38

Smear status	Smear-positive	137	54.37
	Smear-negative	115	45.63

HIV status	Positive	8	2.31
	Negative	339	97.69

Treatment category	New	281	80.98
	Transfer-in (TI)	65	18.73
	Relapse	1	0.29

**Table 2:** The proportion of student TB cases among total students enrolled at Jimma University by academic year, 11 Sep. 2010-10 Sep. 2015 (per 10,000 student population).

Academic year	Total no. of students enrolled	Total no. of student TB cases	The proportion of student TB cases among total students	Total no. of female students enrolled	Total no. of female student TB cases	The proportion of female student TB cases among female students
2010/11	16,061	114	70.98	4,385	17	38.77

2011/12	18,169	121	66.60	4,597	38	82.66

2012/13	18,830	19	10.09	5,348	3	5.61

2013/14	19,433	48	24.70	5,970	17	28.48

2014/15	19,511	45	23.06	6,191	7	11.31

Total	92,004	347	37.72	26,491	82	30.95

## Data Availability

The data used to support the findings of this study are included within the article.
